# Compositional and Bioactive Differentiation of *Opuntia* spp. Fruit Varieties by PCA and LDA

**DOI:** 10.3390/foods14183170

**Published:** 2025-09-11

**Authors:** Liliana Espírito Santo, Cláudia S. G. P. Pereira, Anabela S. G. Costa, Agostinho Almeida, João C. M. Barreira, Maria Beatriz P. P. Oliveira, Ana F. Vinha

**Affiliations:** 1 LAQV/REQUIMTE, Department of Chemical Sciences, Faculty of Pharmacy, University of Porto, R. Jorge de Viterbo Ferreira 228, 4050-313 Porto, Portugal; lilianaespiritosanto81@gmail.com (L.E.S.); claudia.guimaraes.pp@gmail.com (C.S.G.P.P.); beatoliv@ff.up.pt (M.B.P.P.O.);; 2 Nutrition and Bromatology Group, Department of Analytical Chemistry and Food Science, Faculty of Science, University of Vigo, E-32004 Ourense, Spain; 3 Mountain Research Centre (CIMO), ESA, Polytechnic Institute of Bragança, Campus de Santa Apolónia, 5300-253 Bragança, Portugal; 4 FP-I3ID, Research Institute, Innovation and Development Fernando Pessoa, Faculty of Health Sciences, Fernando Pessoa University, Praça 9 Abril 349, 4249-004 Porto, Portugal

**Keywords:** *Opuntia* spp., nutritional composition, mineral profile, bioactive compounds, antioxidant activity, principal component analysis, linear discriminant analysis

## Abstract

The nutritional, mineral, and bioactive profiles of four *Opuntia* fruit varieties—*Opuntia robusta* red variety (OR-RV) and three *Opuntia ficus-indica* varieties (red, yellow, and green: OFI-RV, OFI-YV, and OFI-GV, respectively)—were characterized to assess their compositional diversity and potential discriminant markers. Standard analytical procedures were applied to determine proximate composition, individual sugars, fibre content, mineral concentration, and bioactive compounds, followed by antioxidant activity assays. Principal Component Analysis (PCA) and Linear Discriminant Analysis (LDA) were used to explore multivariate patterns and identify variables with the greatest discriminatory power. Results revealed significant inter-varietal differences across all measured parameters (*p* < 0.05). OR-RV displayed the highest non-fibre carbohydrate, protein, copper, and ascorbic acid contents, as well as superior antioxidant activity. OFI-GV stood out for its high soluble and insoluble fibre, magnesium, and strontium levels, while OFI-YV was characterized by elevated sodium and calcium, and OFI-RV by increased protein and glucose contents. LDA identified ascorbic acid, protein, and five mineral elements (Sr, Zn, Cu, Mn, B) as key discriminant variables, achieving 100% classification accuracy. These findings highlight compositional diversity among *Opuntia* varieties and support their differentiated use in food and health applications.

## 1. Introduction

The Indian fig tree (*Opuntia ficus-indica*) is an exotic Cactaceae family plant that emerged in Central America and has now grown naturalized in the South Africa, India, Australia, and Mediterranean region [1]. Currently, *O. ficus-indica* is a highly valued domesticated cactus in the worldwide agricultural economy in arid and semi-arid regions. In fact, *O. ficus-indica* possesses morphological and physiological characteristics that maximize water efficiency, allowing its spontaneous expansion due to its crassulacean acid metabolism (CAM), which converts more water into dry matter than the C3 and C4 photosynthetic processes [2]. Because of its importance and versatility, this cactus (fruits and cladodes) is now recognized and is currently present in more than 30 countries [3], providing environmental, nutritional, and economic benefits [4,5]. Moreover, recent scientific research has revealed the presence of natural chemicals in cactus fruits that have significant potential for health properties [6,7,8]. Several authors emphasize their nutritional and chemical richness, including vitamins, minerals, polyunsaturated fatty acids, proteins, polysaccharides, carotenoids, and betalains [8,9,10]. Thus, several secondary metabolites, mainly phenolic compounds, including kaempferol 3-*O*-robinobioside-7-*O*-arabinofuranoside, isorhamnetin 3-*O*-galactoside, and isorhamnetin 3-*O*-rhamnoside-7-*O*-(rhamnosyl-hexoside) have been described [11]. Rodrigues et al. [7] reported higher contents of quercetin, isorhamnetin, luteolin, and kaempferol in cactus fruits than other fruit varieties including papaya, banana, and watermelon, which contributes to its high antioxidant activity [12].

Likewise, *Opuntia robusta* is widely distributed being particularly abundant in Mexico, South Africa, and the Mediterranean basin. Its purple-coloured fruit contains phenolic compounds (particularly phenylpropanoic and hydroxycinnamic acids, and flavonoids), vitamins C and D, and pigments (such as betalains, and mainly betacyanins) [13].

*O. ficus-indica* and *O. robusta* had also been highlighted as sources of mineral elements, particularly Al, Ba, Ca, Cu, Fe, K, Mg, Mn, Na, P, and Zn [14,15].

Despite the benefits attributed to both fruits it is important to emphasize that the chemical profile contributes to those beneficial properties and that it can vary depending on several factors, including soil, planting location, weather circumstances, age, and species [16]. Internationally, *Opuntia* spp. are cultivated primarily to produce fruits and/or cladodes intended for fresh consumption or industrial processing. Nowadays, in Portugal, there are several regions with potential for growing *Opuntia* spp. shrubs; however, few information is known about the nutritional and chemical composition of their fruits. This study aimed to evaluate the nutritional and mineral composition, as well as the bioactivity indicators of fruits from different varieties of *O. ficus-indica* and *O. robusta*. Additionally, principal component analysis (PCA) and linear discriminant analysis (LDA) were applied to select the best variety for specific industrial uses.

## 2. Materials and Methods

### 2.1. Material and Sample Preparation

The present study was conducted on fruits of two *Opuntia* species (*O. robusta* and *O. ficus-indica*). Due to colour differences, sampling included (i) red pulp *O. robusta*; (ii) red pulp *O. ficus-indica*; (iii) yellow pulp *O. ficus-indica*; and (iv) green pulp *O. ficus-indica*. Fruits were cultivated under an organic farming system and were harvested in 2023 from orchards located in Torres Novas (39°31′04″ N, 8°30′10″ O). The fruits were manually collected at the optimal stage of maturity, determined by their colour and the ease of glochid detachment, and promptly transported to the laboratory.

Unharmed fruits were washed with tap water to remove impurities and glochids present on their surface, and then manually peeled and stored separately in sample bottles at −80 °C, and subsequently lyophilized (Telstar Cryodos-80 Terrassa, Barcelona, Spain). After lyophilization, all samples were ground in a mill (Grindomix GM200, Rech, Germany) to obtain homogeneous powder samples. All determinations were carried out in triplicate.

### 2.2. Nutritional Analysis

Nutritional assays followed AOAC methods [17]. Fruits were initially evaluated for moisture content, through an infrared balance (Scaltec model SMO01, Scaltec Instruments, Heiligenstadt, Germany) (AOAC, 925.09). Ashes were measured after incineration at 500 °C (AOAC, 935.42). Total lipids were assessed using the Soxhlet methodology (AOAC, 989.05) and crude protein followed the Kjeldahl methodology (AOAC, 991.02). Protein content was calculated based on food nitrogen concentration with a conversion factor of 6.25. The enzymatic–gravimetric technique [17] was used to analyze total and insoluble fibre content. Soluble fibre and total carbohydrates were measured by their differential. The results were presented as g per 100 g of dry weight (dw). The energy value was calculated as follows: Energy value (kcal/100 g) = (g of protein × 4) + (g of fat × 9) + (g of carbohydrates × 4) + (g of fibre × 2) [18].

### 2.3. Determination of Free Sugars by HPLC

Approximately 250 mg of each fruit variety was placed in a Falcon tube and diluted with 10 mL of deionized water. After 30 min of homogenization, the tubes were centrifuged at 5000 rpm for 15 min using a Heraeus Megafuge 16 (Hanau, Germany). Syringe filters were used to filter samples and transfer into injection vials. Free sugar content of each sample was evaluated using an HPLC system (Jasco, Tokyo, Japan) with an evaporative light scattering detector (ELSD), following the protocol described by Montesano et al. [19]. Free sugars were identified based on retention time of recognized standards and measured using calibration curves. The concentrations of the standards ranged from 0.2 to 6.0 mg/mL, for glucose and fructose. The separation was performed on a Shodex Asahipak NH_2_P 50 4E column (4.6 mm I.D. × 250 mm) with an isocratic system and two eluents: 25% water and 75% acetonitrile. Samples were eluted at a flow rate of 0.5 mL/min for 20 min at 30 °C. Results were provided in g/100 g dw.

### 2.4. Elemental Analysis

Mineral analysis was performed in accordance with Pinto et al. methodology [20]. Each fruit (~250 mg) was processed in an MLS-1200 Mega microwave digestion equipment with an HPR-1000/10 S rotor (Milestone, Sorisole, Italy) using 65% nitric acid and 30% hydrogen peroxide solution. After digestion, the resulting solution was diluted with 25 mL of ultrapure water. The macro and trace element contents were determined using Flame Atomic Absorption Spectrometry (FAAS), Perkin Elmer 3100 (Überlingen, Germany). Calibration standards were obtained by diluting standard stock solutions of Ca, Na, Mg, Fe, or K to 1000 mg/L concentrations. The elemental analysis was performed using an iCAP™ Q ICP-MS (Thermo Fisher Scientific, Bremen, Germany). Calibration standards ranging from 0.5 to 200 µg/L were derived using the commercial 10 mg/L PlasmaCAL SCP-33-MS multi-element standard solution. The AccuTrace™ ICP MS-200.8-IS-1 solution (containing 100 mg/L of Sc, Y, In, Tb, and Bi) was diluted to prepare 100 µg/L internal standards solutions. The isotopes detected were 7Li, 9Be, 11B, 27Al, 48Ti, 51V, 52Cr, 55Mn, 59Co, 60Ni, 65Cu, 66Zn, 75As, 82Se, 85Rb, 88Sr, 90Zr, 98Mo, 111Cd, 118Sn, 121S, 133Cs, 137Ba, 182W, 208Pb, and 209Bi. All determinations were performed in triplicate and findings were based on dry weight (dw).

### 2.5. Vitamin C by HPLC

Four grams of material were stabilized with 12 mL of acid solution (10% (*v*/*v*) perchloric acid and 1% (*w*/*v*) metaphosphoric acid in ultrapure water) to stabilize and precipitate proteins in fruit samples. The mixture was thoroughly vortexed for 1 min and diluted in 50 mL with mobile phase solution (Section 2.5.1). After, each sample was filtered twice: first using a 150 mm diameter Macherey-Nalgel filter paper (Macherey-Nalgel GmbH & Co.KG, Düren, Germany) and subsequently through a 0.45 μm Millipore PVDF filter (Millipore Corporation, Bedford, MA, USA). 20 μL was injected into the HPLC apparatus. AA detection was measured at 254 nm [21].

#### 2.5.1. Additional Instrumentation and Chromatographic Conditions

Separation and quantification were performed on an Alliance 2695 HPLC system (Waters, Milford, MA, USA) equipped with a Waters 2996 photodiode array detector (PDA), using a Phenomenex, Synergi™ Hydro-RP (150 × 4.6 mm I.D., 4.0 μm particle size) analytical column from Phenomenex (Torrance, CA, USA) with a SecurityGuard Cartridge AQ C18 (40 × 2.0 mm I.D., 5 μm particle size) from Phenomenex (Torrance, California, USA). The detection signal was obtained, and peak areas were measured and processed using Empower™ software version 2.0 (Waters, Milford, MA, USA). The mobile phase contained 20 mM ammonium dihydrogen phosphate, a pH of 3.5 (adjusted with 85% orthophosphoric acid), and 0.015% (*w*/*v*) metaphosphoric acid. The mobile phase was filtered using a 0.45 μm GH Polypro membrane Pall filter (Gelman Laboratory, Guelph, ON, Canada) and degassed for 30 min. The overall run time was 5 min with a flow rate of 0.6 mL/min. The column temperature was held at 30 °C, and the autosampler at 4 °C. The chromatographic analyses were carried out in triplicate.

### 2.6. Bioactive Contents and Antioxidant Activity

#### 2.6.1. Extract Preparation

To prepare the extraction, approximately 250 mg of fruit were mixed with 50 mL of a 50:50 *v*/*v* hydroalcoholic solution. The mixture was stirred at 40 °C for an hour, following the procedure described by Vinha et al. [22]. Subsequently, the extracts were filtered using Whatman No. 4 filter paper. To analyze bioactive compounds and antioxidant activity, 1 mL of each filtrate solution was kept in 10 mL tubes at −20 °C. All measurements were performed in triplicate.

#### 2.6.2. Total Phenolics and Total Flavonoids Contents

The total phenolics content (TPC) was measured using the Folin–Ciocalteu reagent along with slightly modified procedures for analysis [23]. Succinctly, 150 µL of Folin–Ciocalteu reagent (1:10), 120 µL of Na_2_CO_3_ aqueous solution (7.5% *m*/*v*), and 30 µL of each sample extract was mixed and incubated at 45 °C for 15 min, followed by 30 min of dark incubation at ambient temperature. Absorbance readings at 765 nm were taken using a Synergy HT Microplate Reader (BioTek Instruments, Inc., Winooski, VT, USA). TPC was measured using a calibration curve with gallic acid as a standard (5–100 mg/L; R^2^ = 0.999). Results were represented as mg of gallic acid equivalents (GAE)/g dw. The total flavonoids content (TFC) was calculated using a spectrophotometric assay following previously described methodology [23]. Briefly, 1 mL of each extract was combined with 300 µL of 5% sodium nitrite (NaNO_2_) in 4 mL of distilled water. Upon 5 min at room temperature, 300 µL of 10% AlCl_3_ was added, and after 1 min, 2 mL of sodium hydroxide (NaOH 1 M) and 2.4 mL of distilled water were incorporated. Catechin was used as standard (2.5–400 mg/L; R^2^ = 0.999). The absorbance at 510 nm was evaluated using a Synergy HT Microplate Reader (BioTek Instruments, Inc., Winooski, VT, USA). Results were expressed as mg of catechin equivalents (CE)/g dw.

#### 2.6.3. Antioxidant Activity

Antioxidant capacity was evaluated by the DPPH^∙^ radical scavenging activity and the ferric-reducing antioxidant power, according to the procedures described by Costa et al. [23].

### 2.7. Statistical Analysis

Before the comparative analysis, the normal distribution and the homogeneity of variances were assessed using the Shapiro–Wilk and Levene’s tests, respectively. The statistical tests were applied considering a value of α = 0.05 (95% confidence) using the IBM SPSS Statistics for Windows software version 25.0 (IBM Corp., Armonk, NY, USA). Three independent samples were used in all cases, and each assay was conducted in triplicate. Data were expressed as mean ± standard deviation.

For each *Opuntia* variety (OR-RV, OFI-RV, OFI-YV and OFI-GV), a 1-way analysis of variance (ANOVA) was applied, and statistically different samples were identified with different lowercase superscript letters. For homoscedastic data (*p* > 0.05), Tukey’s honestly significant difference (HSD) test was employed, while for heteroscedastic data, Tamhane’s T2 multiple comparison test was used.

#### 2.7.1. Principal Components Analysis

The distinctiveness of the characterized profiles (nutritional, mineral, and bioactive components) was assessed to determine whether they could effectively differentiate the four *Opuntia* varieties. Three sequential principal component analyses (PCA) were performed. Additionally, analytical parameters showing the largest variations among varieties were identified. The number of dimensions retained was based on eigenvalues (>1), positive Cronbach’s alpha values, and maximized total explained variance. Three dimensions were plotted to facilitate graphical interpretation.

#### 2.7.2. Linear Discriminant Analysis

Furthermore, a linear discriminant analysis (LDA) was employed to identify the variables that most effectively differentiated the fruits of the studied *Opuntia* varieties. Variable selection was performed using a stepwise procedure based on Wilks’ lambda (λ) criterion, applying standard *F*-probability thresholds (*F* = 3.84 for entry and *F* = 2.71 for removal). This method integrates both forward selection and backward elimination steps, ensuring that the statistical significance of previously included variables is reassessed before the addition of new predictors [24]. This method enables the identification of the independent variables that most significantly contributed to the observed differences in the mean score profiles of fruits from each *Opuntia* species. All predictor variables were entered simultaneously, and the standard assumptions underlying linear discriminant analysis (LDA) were thoroughly verified. The significance of the canonical discriminant functions was evaluated using Wilks’ lambda (λ) test. To assess the model’s predictive performance, a leave-one-out cross-validation procedure was applied.

## 3. Results

### 3.1. Nutritional Composition

The proximate composition of the fruits from *O. robusta* and *O. ficus-indica* varieties is shown in Table 1. In fresh weight (fw) basis, moisture stood out as the major component in all cases, reaching the highest value (86.8 ± 0.4 g/100 g fw) in OR-RV, while the lowest was measured in OFI-GV (80.3 ± 0.5 g/100 g fw).

Using dry weight (dw) as reference, the studied fruits consisted of nearly 90% carbohydrates, with a considerable proportion derived from fibre. The highest fibre content was observed in OFI-GV (9.4 ± 0.5 g/100 g dw of soluble fibre and 21.9 ± 0.2 g/100 g dw of insoluble fibre), whereas lowest was found in OR-RV (1.2 ± 0.5 g/100 g dw of soluble fibre and 19.1 ± 0.2 g/100 g dw of insoluble fibre). On the other hand, the highest concentration of non-fibre carbohydrates was recorded in OR-RV (64.7 ± 0.2 g/100 g dw), while the lowest level was detected in OFI-GV (58.2 ± 0.5 g/100 g dw). Protein, the second major nutrient, was significantly higher in the red varieties (OR-RV and OFI-RV), reaching approximately 6.4 g/100 g dw. The studied *Opuntia* fruits were also characterized by low fat levels, ranging from 1.2 ± 0.1 g/100 g dw in OFI-GV to 1.7 ± 0.1 g/100 g dw in OR-RV. Overall, this nutritional profile resulted in an energy value ranging from 328 ± 1 kcal/100 g dw in OFI-GV to 340 ± 1 kcal/100 g dw in OR-RV, consistent with the nutritional profiles reported for other *Opuntia* species [25,26,27].

The ash content (mineral elements are detailed in the next section) was significant, ranging from 3.9 ± 0.1 g/100 g dw (in OFI-RV and OFI-GV) to 6.9 ± 0.2 g/100 g dw in OR-RV.

### 3.2. Mineral Profile

In what concerns minerals, significant (*p* < 0.05) differences were observed for all elements, indicating statistical variability across varieties. Table 2 is structured in four horizontal sections to distinguish macro (g/kg), micro (mg/kg), trace (μg/kg), and ultra-trace (ng/kg) elements, providing a clear overview of the mineral composition.

Regarding the macro elements (quantified in g/kg dw), the four *Opuntia* varieties exhibited significant differences in magnesium (Mg) concentrations, reaching highest values in OFI-GV (2.63 ± 0.05 g/kg), while OR-RV present the lowest (1.00 ± 0.03 g/kg). In particular, these same varieties presented opposite behaviour in what concerns potassium (K) levels, with maximum values in OR-RV (25 ± 2 g/kg), and minimum concentrations in OFI-GV (10.9 ± 0.1 g/kg). The highest calcium (Ca) level was detected in OFI-YV (5.7 ± 0.2 g/kg), with the lowest concentration being quantified in OR-RV (1.8 ± 0.1 g/kg). Overall, these concentrations are consistent with the ranges reported for *Opuntia* species from diverse geographical regions [16,28].

The micro elements (quantified in mg/kg dw) were the ones present in higher number (13 elements in OR-RV and OFI-YV; 12 elements in OFI-RV and OFI-GV). The essential elements included in this group were manganese (Mn: 5.4 ± 0.5 mg/kg in OFI-RV to 63 ± 2 mg/kg in OFI-GV), iron (Fe: 8.4 ± 0.2 mg/kg in OFI-YV to 13.1 ± 0.3 mg/kg in OFI-GV), zinc (Zn: 6.3 ± 0.5 mg/kg in OFI-YV to 14.1 ± 0.1 mg/kg in OFI-GV), copper (Cu: 2.0 ± 0.2 mg/kg in OFI-YV to 8.7 ± 0.3 mg/kg in OR-RV), chromium (Cr: 66 ± 5 mg/kg in OFI-RV and 147 ± 22 mg/kg in OFI-YV—not detected in the remaining varieties), sodium (Na: 2.3 ± 0.3 mg/kg in OR-RV to 5.8 ± 0.3 mg/kg in OFI-YV), and molybdenum (Mo, which was only detected in OR-RV: 1.0 ± 0.1 mg/kg). The other non-essential or toxic elements within the mg/kg range were boron (B: 9.4 ± 0.5 mg/kg in OFI-RV to 34 ± 2 mg/kg in OR-RV), rubidium (Rb: 7.2 ± 0.5 mg/kg in OFI-RV to 29 ± 3 mg/kg in OR-RV), aluminum (Al: 1.3 ± 0.1 mg/kg in OR-RV to 3.4 ± 0.1 mg/kg in OFI-YV; not detected in OFI-RV), titanium (Ti: 1.2 ± 0.1 mg/kg in OR-RV to 4.2 ± 0.1 mg/kg in OFI-YV), nickel (Ni: 0.40 ± 0.05 mg/kg in OFI-YV to 3.2 ± 0.1 mg/kg in OR-RV), strontium (Sr: 0.9 ± 0.1 mg/kg in OFI-RV to 4.5 ± 0.2 mg/kg in OFI-GV), and barium (Ba: 0.8 ± 0.1 mg/kg in OR-RV to 2.1 ± 0.1 mg/kg in OFI-GV). Overall, despite minor variations, the mineral profiles within this concentration range are consistent with those reported for the same or closely related *Opuntia* varieties [25,28].

Regarding the trace elements (detected in the μg/kg range), cobalt (Co), an essential element, was quantified in all samples, ranging from 20 ± 3 μg/kg in OFI-RV to 178 ± 16 μg/kg in OFI-GV. Beryllium (Be) was detected in OFI-GV (20 ± 2 μg/kg) and OR-RV (3.2 ± 0.4 μg/kg), warranting attention, as it is classified as a category 1B carcinogen under the EU Classification, Labelling and Packaging (CLP) Regulation [29], despite the absence of established EU food safety limits.

Among the ultra-trace elements (ng/kg dw), caesium (Cs) was the most abundant, ranging from 5.6 ± 0.5 ng/kg in OFI-RV to 73 ± 3 ng/kg in OFI-GV. Zirconium (Zr) was also present in all varieties, with concentrations from 3 ± 1 ng/kg in OFI-RV to 43 ± 4 ng/kg in OFI-YV. Cadmium (Cd) levels varied between 1.5 ± 0.1 ng/kg in OFI-YV and 2.8 ± 0.2 ng/kg in OR-RV. Additionally, lead (Pb) was detected at comparable concentrations in OR-RV and OFI-GV (28 ng/kg), at a lower level in OFI-YV (6.6 ± 0.3 ng/kg), and was not detected in OFI-RV. Tin (Sn) and tungsten (W) were each found in only one variety, with Sn detected in OFI-YV (53 ± 5 ng/kg) and W in OR-RV (10.9 ± 0.1 ng/kg). Overall, the concentrations of Cd and Pb were several times lower than those reported for Tunisian *Opuntia* varieties [25], and are well below the European Union’s maximum levels for fruits, which are 100 µg/kg for Pb and 50 µg/kg for Cd [30]. Additionally, the European Food Safety Authority (EFSA) has established a tolerable weekly intake (TWI) of 2.5 µg/kg body weight for cadmium, based on dietary exposure and renal effects [31].

Notably, Ca, B, Ti, Fe, Cu, and Zn, were the only elements exhibiting statistically significant differences across all four *Opuntia* varieties. Despite this, the findings indicate substantial variation in mineral composition among the studied genotypes. OFI-RV exhibited the most limited mineral profile, with 19 detected elements, as Al, Mo, Be, Sn, W, and Pb were not detected; in contrast, OR-RV displayed the most widespread profile, comprising 23 elements, with only Cr and Sn as absent elements. OFI-GV was characterized by the presence of 21 elements, lacking Cr, Mo, Sn, and W, whereas OFI-YV contained 22 elements, with the absence of Mo, Be, and W. Notably, none of the *O. ficus-indica* varieties contained detectable levels of Mo or W.

### 3.3. Bioactive Profile

The analyzed *Opuntia* fruits exhibited statistically significant differences (*p* < 0.001) across all measured bioactivity indicators (Table 3). Among them, OR-RV consistently demonstrated the highest values in all assays, with the exception of total phenolic content (750 ± 20 mg GAE/100 g dw), in which it was comparable to OFI-RV (760 ± 32 mg GAE/100 g dw), and dehydroascorbic acid concentration (31 ± 1 mg/100 g dw), which did not differ significantly from that of OFI-GV (32 ± 2 mg/100 g dw). Conversely, OFI-GV showed the lowest levels of total phenolics (606 ± 37 mg GAE/100 g dw) and flavonoids (36 ± 3 mg CE/100 g dw), whereas OFI-RV (26 ± 1 mg/100 g dw) and OFI-YV (28 ± 1 mg/100 g dw) expressed the lowest dehydroascorbic acid concentrations. As already highlighted, ascorbic acid content spiked in OR-RV (304 ± 4 mg/100 g dw), showing a remarkably higher concentration than all remaining varieties (which slightly exceeded 100 mg/100 g dw). In comparison to previously reported values, the total phenolic contents observed in the present study were generally lower, whereas flavonoid concentrations were comparatively higher across the currently analyzed varieties. Ascorbic acid levels, on the other hand, were more consistent with those reported in the literature [7,32]. Overall, these results underscore the superior bioactive profile of the OR-RV variety.

Antioxidant activity, measured using the ferric reducing antioxidant power (FRAP) and DPPH radical scavenging assays, mirrored the trends observed in bioactive compounds’ contents. OR-RV had the highest FRAP value (11,622 ± 792 µmol SFE), significantly (*p* < 0.001) surpassing all other varieties. DPPH values also followed this pattern, with OR-RV demonstrating the strongest activity (192 ± 15 mg TE), and a sequential decrease across OFI-RV, OFI-YV, and OFI-GV. These results seem to indicate an observable correlation between phenolic/flavonoid/ascorbic acid levels and antioxidant capacity in these *Opuntia* species [29,33].

## 4. Discussion

### 4.1. Principal Components Analysis

In the previous sections, differences among the *Opuntia* varieties were compared by analyzing each parameter individually. Although statistically significant differences were observed in the nutritional, mineral, and bioactive profiles, the present analysis intended to verify whether each profile was distinctive enough to differentiate the investigated variety. It should be noted, however that such distinctiveness could be more robust if the samples had been obtained from different regions and harvesting years.

#### 4.1.1. Nutritional Parameters

**Figure 1:**
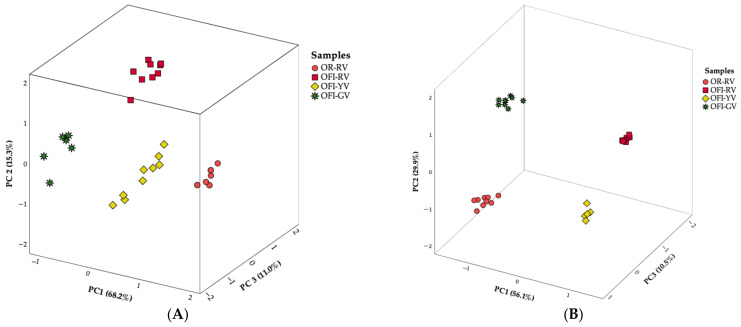
Three-dimensional plots of principal components highlighting nutritional profiles (**A**) and mineral profiles (**B**) differences among *Opuntia* varieties. The percentage of variance explained by each principal component is indicated in the corresponding axis.

Regarding the nutritional parameters (Figure 1A), PCA revealed that the three first components (PC1: Cronbach’s α = 0.948, eigenvalue = 6.824; PC2: Cronbach’s α = 0.386, eigenvalue = 1.532; PC3: Cronbach’s α = 0.104; eigenvalue = 1.103) accounted for 94.5% of the total variance.

PC1 was strongly correlated with moisture and ash, both highest in OR-RV, and negatively correlated with soluble fibre, which was quantified in the lowest concentration in OR-RV; consequently, its markers were the single ones scoring positively in PC1 axis. PC2 was primarily associated with protein and glucose (both highest in OFI-RV) and with insoluble fibre (lowest in OFI-YV), which led to the clear separation of the markers corresponding to OFI-RV and OFI-YV. PC3 highlighted OFI-GV due to its high soluble fibre and low glucose contents, positioning this variety apart from the others.

#### 4.1.2. Mineral Profiles

PCA of the mineral profiles (Figure 1B) showed that the first three dimensions (PC1: Cronbach’s α = 0.967, eigenvalue = 14.014; PC2: Cronbach’s α = 0.902, eigenvalue = 7.483; PC3: Cronbach’s α = 0.646; eigenvalue = 2.634) explained 96.5% of the total variance. OFI-YV emerged as the most distinct variety on PC1, mainly as a result of its low levels of rubidium, cadmium, manganese, and zinc, and its notably high sodium content. PC2 separated OFI-GV due to strong positive correlations with barium, magnesium, and beryllium, all showing maximum valued in this variety. PC3 was mainly associated with aluminum, tin, and zirconium, effectively individualizing OFI-RV, which exhibited minimal concentrations of these elements.

#### 4.1.3. Bioactive Profile

**Figure 2:**
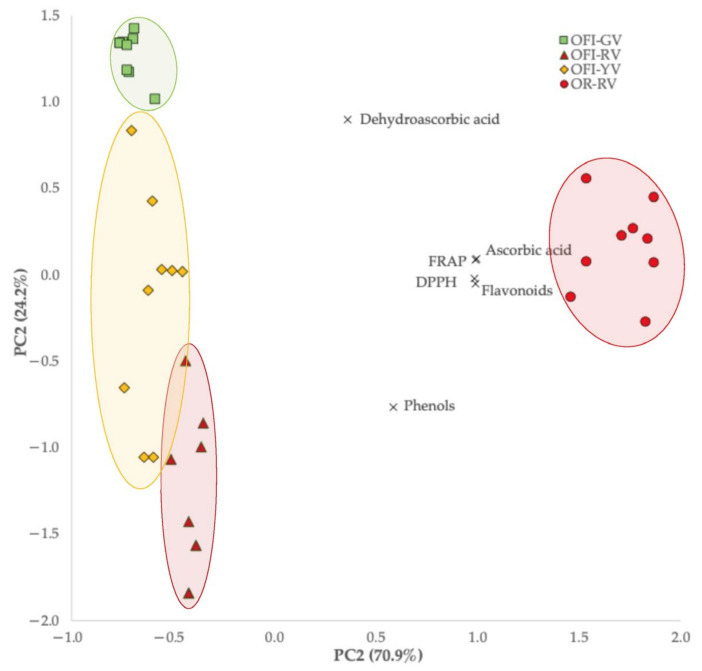
Biplot of objects (*Opuntia* varieties) and principal components (bioactive parameters).

For the bioactive compounds, only the first two PCA dimensions were considered (Figure 2), as the third one yielded a negative Cronbach’s. Together, PC1 (Cronbach’s α = 0.918, eigenvalue = 4.254) and PC2 (Cronbach’s α = 0375, eigenvalue = 1.454) accounted for 95.1% of the total variance. PC1 was primarily correlated with DPPH, flavonoids, FRAP, and ascorbic acid, all of which peaked in OR-RV, making it the most bioactive variety. PC2 was predominantly correlated with dehydroascorbic acid and phenols (−0.634), leading to a clear separation of OFI-GV, mainly due to its lower phenol content.

Overall, OR-RV was primarily characterized by elevated levels of DPPH, flavonoids, FRAP, and ascorbic acid, high moisture and ash contents, and low soluble fibre concentrations; in turn, OFI-RV was mostly characterized by its high levels of glucose and protein, and low levels of aluminum, tin, and zirconium, while OFI-YV exhibited the lowest levels of insoluble fibre, rubidium, cadmium, manganese, and zinc levels, but also the highest concentration in sodium; finally, OFI-GV stood out for its high levels of soluble fibre, glucose, barium, magnesium, and beryllium, along with its notably low phenol content.

### 4.2. Linear Discriminant Analysis

As shown in Table 1, Table 2 and Table 3 and Figure 1 and Figure 2, several parameters exhibited statistically significant differences among the fruits of the studied *Opuntia* varieties. Accordingly, it is pertinent to identify which of the evaluated parameters have the greatest discriminant power when analyzed simultaneously.

Therefore, the results were evaluated using LDA applied to the full data matrix (1260 values) composed of 36 lines (4 cultivars × 3 samples × 3 analysis) × 35 columns (only parameters common to all cultivars were considered). Significant independent variables were identified through a stepwise LDA, based on Wilks’ λ criterion, retaining only those variables that demonstrated statistically significant discriminant ability (*p* < 0.05).

**Figure 3:**
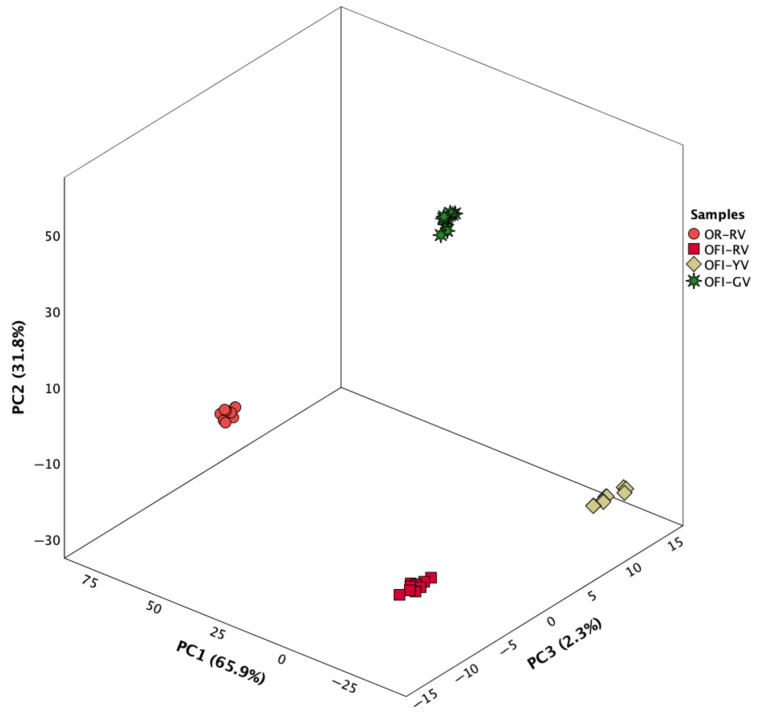
Canonical discriminant functions coefficients defined from nutritional, mineral and bioactive profiles to highlight differences among *Opuntia* varieties.

The three discriminant functions plotted in Figure 3 collectively accounted for 100.0% of the total variance observed in the dataset, with the first function explaining 65.9%, the second 31.8% and the third 2.3%). Out of the 35 initially considered variables, only 7 demonstrated statistically significant discriminant power: ascorbic acid, Sr, Zn, Cu, Mn, B, and protein content.

Discriminant function 1 effectively distinguished OR-RV variety from the others, primarily due to its strong positive correlation with ascorbic acid and copper (an essential element) concentrations, both markedly elevated in this cultivar. Discriminant function 2, which was most strongly associated with strontium and manganese levels, individualized OFI-GV variety, characterized by high concentrations of these two elements, which advises for a moderate consumption of its fruits (particularly due to Sr values). In contrast, the separation between OFI-RV and OFI-YV was achieved primarily through discriminant function 3, which was predominantly ruled by differences in Zr (highest concentration in OFI-YV) and protein (minimum level in OFI-YV) content—the variables with the highest correlations with this function.

The classification accuracy was 100% for both the original grouping of samples and the cross-validation procedure, indicating excellent model performance and robustness.

## 5. Conclusions

The comprehensive characterization of *Opuntia robusta* red variety (OR-RV) and *Opuntia ficus-indica* green, red and yellow varieties revealed clear compositional and functional differences across nutritional, mineral, and bioactive profiles. Among the studied cultivars, OR-RV consistently demonstrated superior attributes (e.g., moisture, protein, copper, and ascorbic acid content, and antioxidant activity values), making it the most promising alternative for health-promoting applications. In contrast, OFI-GV stood out for its elevated fibre, magnesium, and manganese contents, but the lowest antioxidant activity, which might be associated with its lower levels of phenolic compounds, with the additional caveat of containing the highest strontium concentrations. While rich in calcium and sodium, OFI-YV displayed comparatively lower protein and trace element levels, whereas OFI-RV was characterized by higher glucose and protein contents with a favourable lower level of potentially toxic metals.

Multivariate analysis (PCA and LDA) confirmed that each variety possesses a distinct compositional fingerprint. Seven parameters—ascorbic acid, protein, boron, copper, zinc, manganese, and strontium—were enough to achieve 100% accurate classification among varieties, underscoring their value as discriminant biomarkers. Notably, while all varieties offered beneficial nutrient and mineral profiles within recommended safety levels, the elevated strontium in OFI-GV suggests a need for moderated consumption.

Taken together, these findings highlight both the nutritional potential and diversity of *Opuntia* fruits, suggesting opportunities for their targeted application in functional foods, nutraceuticals, and health-promoting diets. Future studies should expand on these results by (i) evaluating the bioavailability and physiological effects of key nutrients and bioactives in vivo, (ii) assessing the long-term health implications of consuming mineral-rich varieties such as OFI-GV, and (iii) exploring breeding and cultivation strategies to optimize desirable traits for specific consumer and industrial needs.

## Figures and Tables

**Table 1:** Moisture content (g/100 g fresh weight), proximate composition (g/100 g dry weight) and energy value for the studied *Opuntia* varieties. The results are presented as mean ± SD.

	*Opuntia* Variety ^1^				Levene’s Test *p*-Value (n = 36)
	OR-RV	OFI-RV	OFI-YV	OFI-GV	
Moisture	86.8 ± 0.4 ^a^	82.1 ± 0.5 ^b^	81.7 ± 0.5 ^b^	80.3 ± 0.5 ^c^	0.766
Fat	1.7 ± 0.1 ^a^	1.5 ± 0.1 ^b^	1.3 ± 0.1 ^c^	1.2 ± 0.1 ^d^	0.004
Protein	6.3 ± 0.1 ^a^	6.4 ± 0.2 ^a^	4.9 ± 0.2 ^c^	5.4 ± 0.2 ^b^	0.286
Non-fibre carbohydrates	64.7 ± 0.2 ^a^	59.5 ± 0.5 ^c^	61.8 ± 0.5 ^b^	58.2 ± 0.5 ^d^	0.003
Fructose	19.6 ± 0.3 ^c^	32.4 ± 0.4 ^a^	32.5 ± 0.5 ^a^	30.7 ± 0.4 ^b^	0.121
Glucose	45 ± 1 ^ab^	46 ± 1 ^a^	43 ± 2 ^b^	41 ± 1 ^c^	0.001
Soluble fibre	1.2 ± 0.1 ^c^	7.7 ± 0.5 ^b^	9.1 ± 0.4 ^a^	9.4 ± 0.5 ^a^	<0.001
Insoluble fibre	19.1 ± 0.1 ^c^	21.0 ± 0.2 ^b^	18.7 ± 0.4 ^d^	21.9 ± 0.2 ^a^	<0.001
Ash	6.9 ± 0.1 ^a^	3.9 ± 0.1 ^c^	4.2 ± 0.1 ^b^	3.9 ± 0.1 ^c^	0.008
Energy	340 ± 1 ^a^	334 ± 1 ^b^	334 ± 1 ^b^	328 ± 1 ^c^	0.011

^1^ Differences among varieties were significant (*p*-value < 0.001) for all parameters; accordingly, samples were evaluated and classified (in each line different lowercase superscript letters identify statistically different values) using Tukey’s HSD (homoscedastic distribution) or the Tamhane’s T2 (heteroscedastic distribution) multiple comparison tests.

**Table 2:** Mineral profile in dry weight basis of the studied Opuntia varieties. The results are presented as mean ± SD ^1^.

	*Opuntia* Variety				Levene’s Test *p*-Value (n = 36)
Minerals	OR-RV	OFI-RV	OFI-YV	OFI-GV	
Macro elements (g/kg)					
Mg	1.00 ± 0.03 ^c^	1.24 ± 0.05 ^b^	1.35 ± 0.03 ^b^	2.63 ± 0.05 ^a^	<0.001
K	25 ± 2 ^a^	12.7 ± 0.4 ^b^	13.7 ± 0.2 ^b^	10.9 ± 0.1 ^c^	<0.001
Ca	1.8 ± 0.1 ^d^	4.5 ± 0.3 ^b^	5.7 ± 0.2 ^a^	4.2 ± 0.1 ^c^	0.009
Micro elements (mg/kg)					
B	34 ± 2 ^a^	9.4 ± 0.5 ^d^	14.3 ± 0.2 ^b^	11.6 ± 0.5 ^c^	<0.001
Na	2.3 ± 0.3 ^c^	3.9 ± 0.4 ^b^	5.8 ± 0.3 ^a^	2.7 ± 0.3 ^c^	0.621
Al	1.3 ± 0.1 ^b^	nd	3.4 ± 0.1 ^a^	1.4 ± 0.2 ^b^	<0.001
Ti	1.2 ± 0.1 ^d^	3.2 ± 0.2 ^b^	4.2 ± 0.1 ^a^	2.9 ± 0.1 ^c^	0.009
Cr	nd	66 ± 5	147 ± 22	nd	<0.001
Mn	46 ± 2 ^b^	5.4 ± 0.1 ^c^	4.1 ± 0.4 ^c^	63 ± 2 ^a^	<0.001
Fe	10.9 ± 0.5 ^b^	9.5 ± 0.5 ^c^	8.4 ± 0.2 ^d^	13.1 ± 0.3 ^a^	0.046
Ni	3.2 ± 0.1 ^a^	0.50 ± 0.04 ^c^	0.40 ± 0.05 ^c^	1.3 ± 0.1 ^b^	0.028
Cu	8.7 ± 0.3 ^a^	2.5 ± 0.1 ^b^	2.0 ± 0.2 ^d^	2.2 ± 0.1 ^c^	<0.001
Zn	12.3 ± 0.4 ^b^	7.3 ± 0.5 ^c^	6.3 ± 0.5 ^d^	14.1 ± 0.1 ^a^	<0.001
Rb	29 ± 3 ^a^	7.2 ± 0.5 ^c^	6.9 ± 0.2 ^c^	22 ± 1 ^b^	<0.001
Sr	1.8 ± 0.1 ^b^	0.9 ± 0.1 ^c^	0.8 ± 0.1 ^c^	4.5 ± 0.2 ^a^	0.019
Mo	1.0 ± 0.1	nd	nd	nd	-
Ba	0.8 ± 0.1 ^c^	1.2 ± 0.1 ^b^	1.2 ± 0.1 ^b^	2.1 ± 0.1 ^a^	0.061
Trace elements (μg/kg)					
Be	3.2 ± 0.4	nd	nd	20 ± 2	0.012
Co	113 ± 4 ^b^	20 ± 3 ^c^	30 ± 3 ^c^	178 ± 16 ^a^	<0.001
Zr	3 ± 1 ^b^	4 ± 1 ^b^	43 ± 4 ^a^	5 ± 1 ^b^	<0.001
Cd	2.8 ± 0.2 ^a^	1.7 ± 0.1 ^c^	1.5 ± 0.2 ^c^	2.5 ± 0.1 ^b^	0.159
Sn	nd	nd	53 ± 5	nd	-
Cs	19 ± 2 ^b^	5.6 ± 0.5 ^c^	5.5 ± 0.4 ^c^	73 ± 3 ^a^	<0.001
W	10.9 ± 0.1	nd	nd	nd	-
Pb	28 ± 4 ^a^	nd	6.6 ± 0.3 ^b^	28 ± 2 ^a^	<0.001

^1^ Differences among varieties were significant (*p*-value < 0.001) for all parameters; accordingly, samples were evaluated and classified (in each line different lowercase superscript letters identify statistically different values) using Tukey’s HSD (homoscedastic distribution) or the Tamhane’s T2 (heteroscedastic distribution) multiple comparison tests. For beryllium and chromium, values were compared through a Student’s *t*-test.

**Table 3:** Bioactive compounds (per 100 g dw) characterized in the studied *Opuntia* varieties. The results are presented as mean ± SD.

	*Opuntia* Variety ^1^				Levene’s Test *p*-Value (n = 36)
	OR-RV	OFI-RV	OFI-YV	OFI-GV	
**Phenols (mg GAE)**	750 ± 20 ^a^	760 ± 32 ^a^	679 ± 30 ^b^	606 ± 37 ^c^	0.185
**Flavonoids (mg CE)**	136 ± 11 ^a^	68 ± 4 ^b^	50 ± 3 ^c^	36 ± 4 ^d^	0.002
**FRAP (μmol SFE)**	11,622 ± 792 ^a^	4066 ± 274 ^b^	3741 ± 352 ^b^	2892 ± 122 ^c^	0.006
**DPPH (mg TE)**	192 ± 15 ^a^	98 ± 5 ^b^	80 ± 10 ^c^	51 ± 4 ^d^	0.018
**Dehydroascorbic acid (mg)**	31 ± 1 ^a^	26 ± 1 ^b^	28 ± 1 ^b^	32 ± 2 ^a^	0.082
**Ascorbic acid (mg)**	304 ± 4 ^a^	108 ± 2 ^c^	123 ± 1 ^b^	110 ± 3 ^c^	0.120

^1^ Differences among varieties were significant (*p*-value < 0.001) for all parameters; accordingly, samples were evaluated and classified (in each line lowercase superscript letters identify statistically different values) using Tukey’s HSD (homoscedastic distribution) or the Tamhane’s T2 (heteroscedastic distribution) multiple comparison tests.

## Data Availability

The original contributions presented in this study are included in the article. Further inquiries can be directed to the corresponding author.
